# The clinical value of lncRNA NEAT1 in digestive system malignancies: A comprehensive investigation based on 57 microarray and RNA-seq datasets

**DOI:** 10.18632/oncotarget.14756

**Published:** 2017-01-19

**Authors:** Dan-Dan Xiong, Zhen-Bo Feng, Wei-Luan Cen, Jing-Jing Zeng, Lu Liang, Rui-Xue Tang, Xiao-Ning Gan, Hai-Wei Liang, Zu-Yun Li, Gang Chen, Dian-Zhong Luo

**Affiliations:** ^1^ Department of Pathology, First Affiliated Hospital of Guangxi Medical University, Nanning, Guangxi, Zhuang, China

**Keywords:** LncRNA, NEAT1, digestive system malignancies, clinical value, microarray and RNA-seq datasets

## Abstract

This comprehensive investigation was performed to evaluate the expression level and potential clinical value of NEAT1 in digestive system malignancies. A total of 57 lncRNA datasets of microarray or RNA-seq and 5 publications were included. The pooled standard mean deviation (SMD) indicated that NEAT1 was down-regulated in esophageal carcinoma (ESCA, SMD = −0.35, 95% CI: −0.5~-0.20, *P* < 0.0001) and hepatocellular carcinoma (HCC, SMD = −0.47, 95% CI: −0.60~-0.34, *P* < 0.0001), while in pancreatic cancer (PC), NEAT1 was up-regulated (SMD = 0.45, 95% CI: 0.2~0.71, *P* = 0.001). However, NEAT1 expression in gastric cancer (GC), colorectal cancer (CRC), biliary tract cancer (BTC) and gallbladder carcinoma (GBC) showed no significant difference between cancer and control groups. The pooled area under the curve values for ESCA, GC, CRC, PC and HCC were 0.60, 0.89, 0.81, 0.77 and 0.69, respectively. Furthermore, our result demonstrated that a high expression of NEAT1 predicted an unfavorable prognosis in patients with digestive system malignancies (HR: 1.50, 95% CI: 1.28-1.76, *P* < 0.0001). Our study suggests that NEAT1 may play different roles in the initiation and progression of digestive system cancers and could be a potential diagnostic and prognostic biomarker in patients with digestive system carcinomas. Further and stricter studies with a larger number of cases are necessary to strengthen our conclusions.

## INTRODUCTION

With a complex physiology and anatomy composed of many ducts and glands, the digestive system has been found to be involved in the pathogenesis of many diseases, especially cancers. The most prevalent digestive system malignancies, colorectal cancer (CRC), hepatocellular carcinoma (HCC), gastric cancer (GC), esophageal cancer (ESCA), pancreatic cancer (PC), gallbladder carcinoma (GBC) and biliary tract cancer (BTC), have been identified as the third, fifth, sixth, ninth, fourteenth and twentieth common cancers worldwide, respectively. According to recent cancer statistics, the prevalence and mortality rate of digestive system malignancies are increasing. The mortality of GC, HCC, CRC, ESCA, PC, GBC and BTC were ranked second, third, fourth, sixth, eighth and fifteenth for cancer mortality worldwide in 2013, respectively [1]. Therefore, it is necessary to explore novel effective molecular biomarkers to diagnose digestive cancer patients early and precisely and screen for more effective therapeutic targets to protect patients from mortality associated with digestive malignancies or at least to prolong their lifespan.

Long non-coding RNAs (LncRNAs) are non-protein coding RNA molecules greater than 200 nucleotides in length. Accumulating evidence demonstrates that lncRNAs may be involved in various cell signal pathways and act as either oncogenes or tumor suppressors. Therefore, lncRNAs may have complex and comprehensive functions in the carcinogenesis and development of human malignancies [2–4]. Evidence presented in previous studies suggests that lncRNAs play vital roles in tumor cell growth, proliferation, differentiation and apoptosis, as well as chromosome inactivation, nuclear domain organization and post-transcriptional gene regulation [5–10]. Additionally, lncRNAs have been used as novel biomarkers and therapeutic targets for various cancers [11, 12].

Nuclear paraspeckle assembly transcript 1 (NEAT1) is a lncRNA that encodes two variants of NEAT1_v1 (3.7 kb) and NEAT1_v2 (23 kb) [13]. It is exclusively localized in a subnuclear structure called a paraspeckle and serves as an architectural component of the paraspeckle [14–17]. NEAT1 participates in the process of gene expression regulation by retaining and editing mRNAs in the nucleus [18]. This association suggests that NEAT1 might play a critical role in the regulation of gene expression and consequent physiological and pathophysiological processes [19, 20]. However, studies on the role of NEAT1 in human malignancies have remained limited until now. A recent meta-analysis conducted by Yang et al. [21] showed that an elevated expression of NEAT1 indicated a worse prognosis in cancer patients. However, the prognostic value of NEAT1 in patients with digestive system carcinomas had not been presented separately. It is worth mentioning that there is no systematic report on the expression level and diagnostic role of NEAT1 in digestive system cancers.

Therefore, we performed the present systematic study of available lncRNA expression data based on high-throughput data from the Gene Expression Omnibus (GEO), ArrayExpress, Oncomine, and The Cancer Genome Atlas (TCGA) and validated data from literature related to the prognostic significance of NEAT1 to evaluate the expression pattern and potential clinical value of NEAT1 in digestive system malignancies.

## RESULTS

### Expression level of NEAT1 in digestive system malignancies

**Figure 1:**
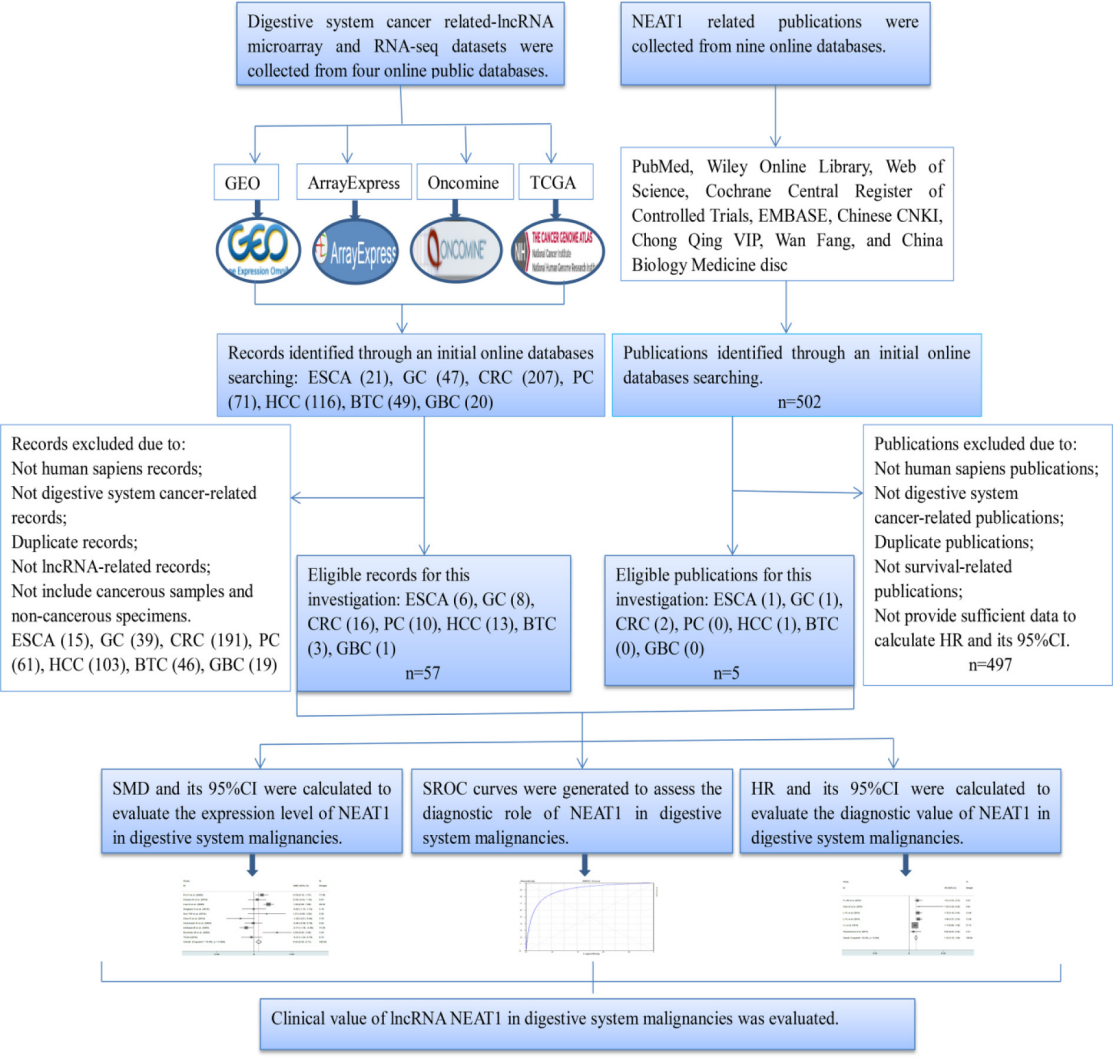
Flow chart of the study design in this investigation

The detailed dataset selection procedure is presented in Figure 1. A total of 57 lncRNA expression datasets including six for ESCA, eight for GC, sixteen for CRC, ten for PC, thirteen for HCC, three for BTC and one for GBC were included in this study. The characteristics of all of the included datasets are shown in Table 1.

**Table 1:** Characteristics of microarray and RNA-seq datasets included in the study

Number	First author (publication year)	Country	Cancer type	Sample source	Data source	Platform	Cancer group	Normal controls	Mean1±SD1	Mean0±SD0
1	Kimchi ET et al. (2004)	USA	ESCA	Tissue	GEO: GSE1420	GPL96	8	16	8.22±1.19	8.36±0.91
2	Hu N et al. (2011)	USA	ESCA	Tissue	GEO: GSE20347	GPL571	17	17	6.38±1.27	7.34±0.61
3	Li J et al. (2014)	China	ESCA	Tissue	GEO: GSE53625	GPL18109	179	179	14.35±0.70	14.67±0.67
4	Su H et al. (2010)	China	ESCA	Tissue	GEO: GSE23400	GPL96 GPL97	104	104	10.89±1.67	11.16±1.43
5	Hippo Y et al. (2005)	Japan	GC	Tissue	GEO: GSE2685	GPL80	22	8	3.64±1.59	4.67±1.39
6	Hu Y et al. (2014)	China	GC	Tissue	GEO: GSE50710	GPL13825	10	10	15.66±0.73	16.11±0.38
7	Gu W et al. (2013)	China	GC	Tissue	GEO: GSE53137	GPL15314	6	6	15.41±1.25	15.0±0.65
8	Aaltonen LA et al. (2010)	Finland	CRC	Tissue	GEO: GSE24514	GPL96	34	15	6.42±0.94	6.65±1.01
9	Hong Y et al. (2007)	Singapore	CRC	Tissue	GEO: GSE4107	GPL570	12	10	12.94±0.94	12.77±0.94
10	Ahmed K et al. (2012)	Japan	CRC	Tissue	GEO: GSE32323	GPL570	17	17	12.15±0.65	12.64±0.58
11	Brunner AL et al. (2012)	USA	CRC	Tissue	GEO: GSE28866	GPL10999	3	3	9.59±0.36	10.34±0.75
12	Nielsen MM et al. (2016)	Denmark	CRC	Tissue	GEO: GSE76713	GPL16228	44	20	8.35±1.43	8.71±1.75
13	Pei H et al. (2009)	USA	PC	Tissue	GEO: GSE16515	GPL570	36	16	12.71±0.66	12.19±0.73
14	Hiraoka N et al. (2010)	Japan	PC	Tissue	GEO: GSE19650	GPL570	9	13	15.68±0.63	15.49±0.62
15	Liviu B et al. (2009)	Romania	PC	Tissue	GEO: GSE15471	GPL570	39	39	12.40±0.33	11.67±0.67
16	Sergeant G et al. (2012)	Belgium	PC	Tissue	GEO: GSE18670	GPL570	6	6	12.02±0.81	12.03±0.46
17	Sun YW et al. (2014)	China	PC	Tissue	GEO: GSE57144	GPL13825	3	3	15.56±0.51	15.01±0.39
18	Dong R et al. (2013)	China	HCC	Tissue	GEO: GSE51701	GPL17843	4	4	11.48±0.80	12.71±0.43
19	Yang F et al. (2011)	China	HCC	Tissue	GEO: GSE27462	GPL11269	5	5	11.77±0.48	11.96±0.49
20	Gao Y et al. (2015)	China	HCC	Tissue	GEO: GSE67260	GPL19072	10	5	3.38±0.86	3.94±0.49
21	Xu X et al. (2014)	China	HCC	Tissue	GEO: GSE61850	GPL19243	5	5	2.97±0.37	3.05±0.59
22	Cao C et al. (2014)	China	HCC	Tissue	GEO: GSE58043	GPL13825	7	7	14.46±1.27	14.60±0.55
23	Fu H et al. (2014)	China	HCC	Tissue	GEO: GSE55191	GPL15314	3	3	7.52±1.09	9.51±1.35
24	Wang K et al. (2013)	China	HCC	Tissue	GEO: GSE49713	GPL11296	5	5	12.66±1.50	14.21±0.34
25	Sulpice L et al. (2013)	France	BTC	Tissue	GEO: GSE45001	GPL14550	6	6	4.87±2.16	6.41±1.44
26	Xu X et al. (2014)	China	BTC	Tissue	GEO: GSE61850	GPL19243	5	5	6.05±0.70	6.15±1.11
27	Wang J et al. (2015)	China	GBC	Tissue	GEO: GSE74048	GPL20115	3	3	2.86±0.92	3.34±0.21
28	Wang B et al. (2016)	China	GC	Tissue	ArrayExpress: E-GEOD-84787	GPL17077	10	10	13.79±3.18	13.59±4.14
29	Frierson H Jr et al. (2016)	USA	CRC	Tissue	ArrayExpress: E-GEOD-77953	GPL96	45	13	5.54±0.75	6.15±1.28
30	Lin G et al. (2012)	USA	CRC	Tissue	ArrayExpress: E-GEOD-41328	GPL570	10	10	8.13±0.50	8.08±0.45
31	Hong Y et al. (2010)	Singapore	CRC	Tissue	ArrayExpress: E-GEOD-9348	GPL570	70	12	12.35±0.85	12.84±0.52
32	Shi X et al. (2015)	China	CRC	Tissue	ArrayExpress: E-GEOD-41657	GPL6480	19	12	11.52±4.68	8.70±2.87
33	Wang Q et al. (2011)	Germany	CRC	Tissue	ArrayExpress: E-GEOD-31905	GPL6480	38	7	6.05±2.82	5.89±0.94
34	Chen R et al. (2014)	China	PC	Tissue	ArrayExpress: E-GEOD-61166	GPL16956	8	4	8.77±0.61	9.47±0.35
35	Kao KJ et al. (2014)	China	HCC	Tissue	ArrayExpress: E-GEOD-60502	GPL96	18	18	10.27±1.17	11.03±0.33
36	Villa E et al. (2014)	Italy	HCC	Tissue	ArrayExpress: E-GEOD-54236	GPL6480	81	80	11.91±1.18	12.23±0.80
37	Roessler S et al. (2010)	USA	HCC	Tissue	ArrayExpress: E-GEOD-14520	GPL571 GPL3921	247	239	5.80±1.21	7.09±1.05
38	Wang S et al. (2006)	USA	ESCA	Tissue	Oncomine	NR	7	17	4.90±2.39	6.15±2.27
39	Chen X et al. (2003)	USA	GC	Tissue	Oncomine	NR	98	18	5.40±2.54	4.26±1.33
40	D'Errico M et al. (2010)	Italy	GC	Tissue	Oncomine	GPL570	38	31	12.96±0.61	13.60±0.44
41	Wang Q et al. (2010)	China	GC	Tissue	Oncomine	GPL570	12	15	14.16±0.52	14.29±0.34
42	Zhou TT et al. (2002)	USA	CRC	Tissue	Oncomine	NR	9	8	7.22±2.71	8.33±2.41
43	Kaiser S et al. (2006)	USA	CRC	Tissue	Oncomine	GPL570	100	5	11.07±0.89	10.84±0.37
44	Skrzypczak M et al. (2010)	Poland	CRC	Tissue	Oncomine	GPL570	101	44	8.37±1.80	8.64±1.46
45	Ki DH et al. (2007)	South Korea	CRC	Tissue	Oncomine	GPL4811	68	28	5.37±1.90	4.57±1.11
46	Gaspar C et al. (2008)	Portugal	CRC	Tissue	Oncomine	GPL3408	10	44	2.37±0.92	3.11±1.35
47	Grutzmann R et al. (2004)	Germany	PC	Tissue	Oncomine	A-AFFY-33A-AFFY-34	14	9	4.68±0.84	4.73±0.91
48	Ishikawa M et al. (2005)	Japan	PC	Tissue	Oncomine	GPL96GPL97	18	21	5.77±1.79	6.98±1.53
49	Buchholz M et al. (2005)	Germany	PC	Tissue	Oncomine	NR	8	6	8.78±1.3	6.19±0.98
50	Wurmbach E et al. (2007)	USA	HCC	Tissue	Oncomine	GPL570	35	10	12.38±0.77	12.12±0.82
51	Mas VR et al. (2007)	USA	HCC	Tissue	Oncomine	GPL96 GPL571	64	19	5.81±0.83	5.59±0.97
52	TCGA (2016)	USA	ESCA	Tissue	TCGA	None	162	11	15.04±0.9	14.85±0.81
53	TCGA (2016)	USA	GC	Tissue	TCGA	None	375	32	14.4±1.16	13.47±1.31
54	TCGA (2016)	USA	CRC	Tissue	TCGA	None	647	51	13.16±1.56	13.27±0.90
55	TCGA (2016)	USA	PC	Tissue	TCGA	None	178	4	14.45±1.22	14.73±0.59
56	TCGA (2016)	USA	HCC	Tissue	TCGA	None	374	50	13.32±1.20	12.36±1.24
57	TCGA (2016)	USA	BTC	Tissue	TCGA	None	36	9	14.19±0.84	13.6±0.71

ESCA: esophageal cancer; GC: gastric carcinoma; CRC: colorectal cancer; PC: pancreatic cancer; HCC: hepatocellular carcinoma; GBC: gallbladder carcinoma; BTC: biliary tract cancer; NR: data not report; SD: standard deviation; Mean1±SD1: expression level of NEAT1 in cancer group; Mean0±SD0: expression level of NEAT1 in normal control group

**Figure 2:**
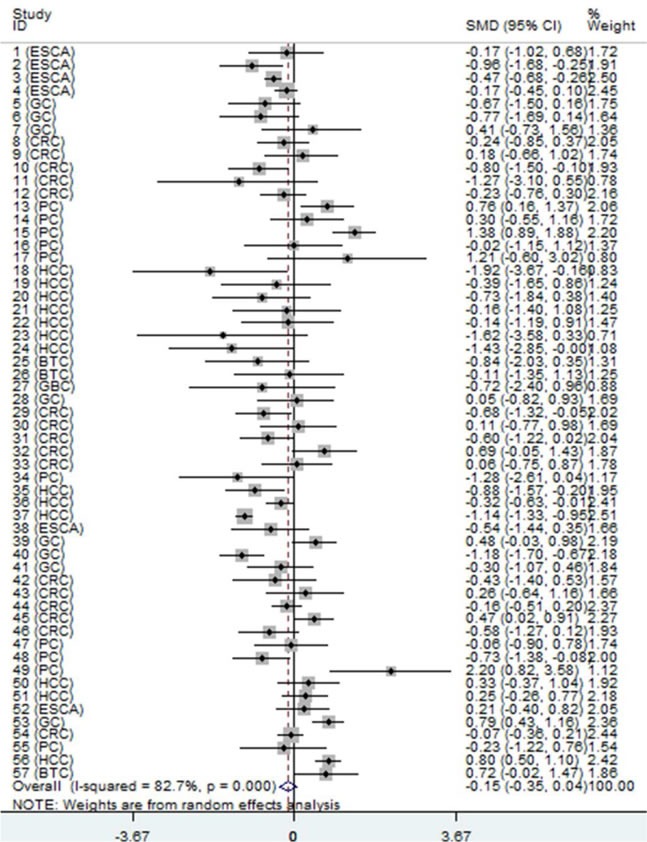
Forest plot of datasets evaluating NEAT1 expression between digestive system cancer and normal control groups (random-effects model) SMD > 0 indicates that NEAT1 expression was higher in cancerous specimens than that in non-cancerous samples. Each horizontal line represents an individual study. The middle point and the length of the two horizontal lines represent the SMD and the 95% CI of each individual study, respectively. The diamond indicates the overall SMD and corresponding 95% CI. The middle vertical line is an invalid line.

**Figure 3:**
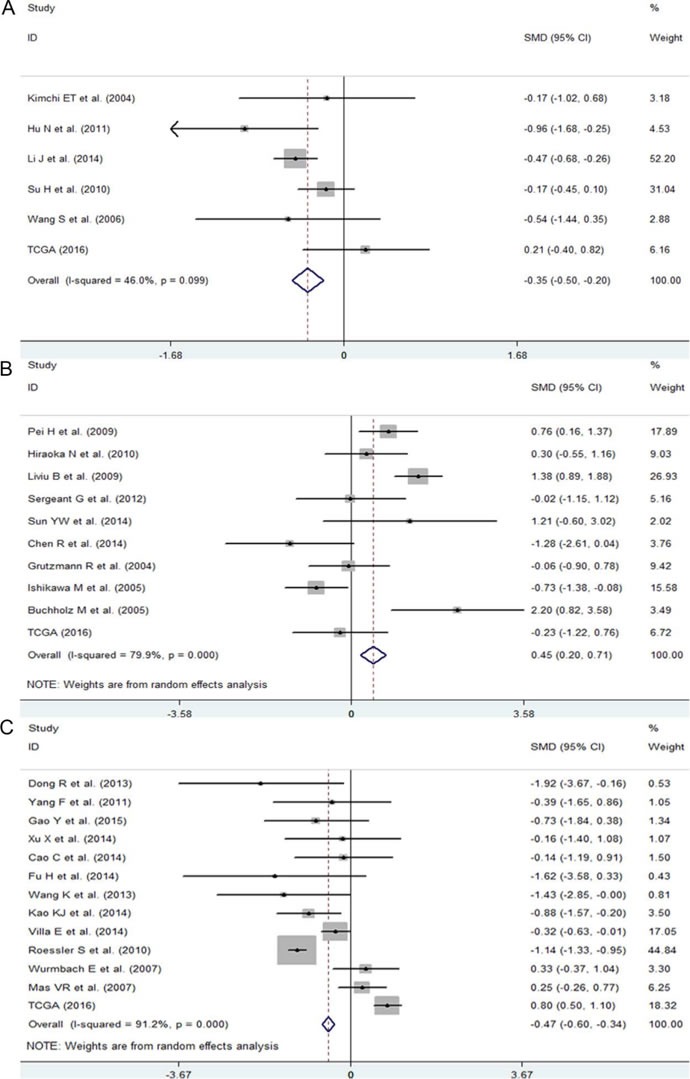
The expression level of NEAT1 in ESCA, PC and HCC **A**. Forest plot of datasets evaluating NEAT1 expression between ESCA and normal control groups. **B**. Forest plot of datasets evaluating NEAT1 expression between ESCA and normal control groups. **C**. Forest plot of datasets evaluating NEAT1 expression between ESCA and normal control groups.

**Figure 4:**
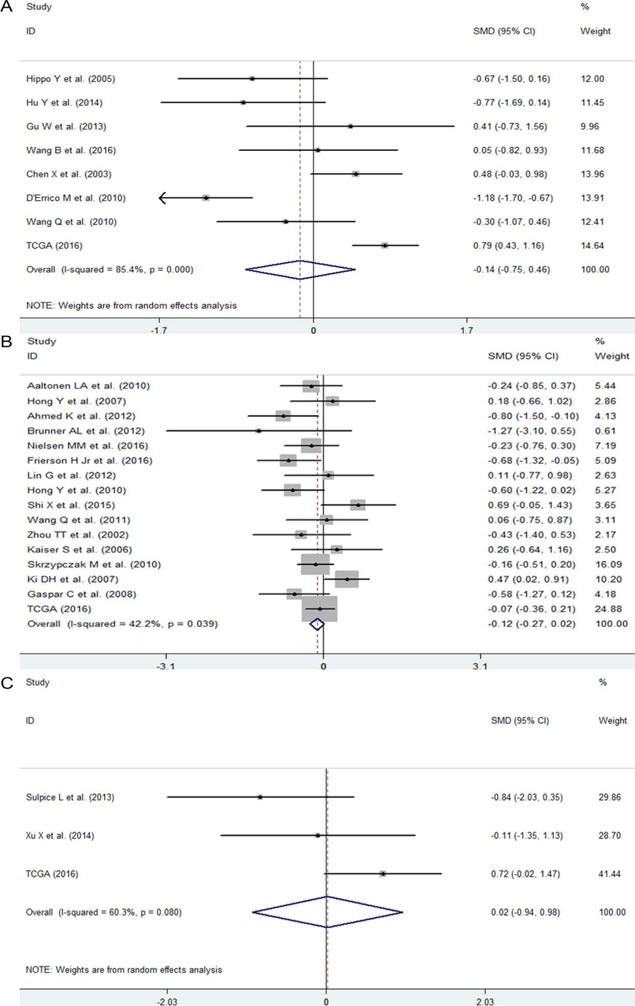
The expression level of NEAT1 in GC, CRC and BTC **A**. Forest plot of datasets evaluating NEAT1 expression between GC and normal control groups. **B**. Forest plot of datasets evaluating NEAT1 expression between CRC and normal control groups. **C**. Forest plot of datasets evaluating NEAT1 expression between BTC and normal control groups.

First, we evaluated the expression level of NEAT1 in digestive system malignancies in the 57 high-throughput datasets of microarray or RNA-seq. A random-effects model was selected to calculate the pooled standard mean deviation (SMD) and 95% confidence interval (95% CI) because noticeable heterogeneity was observed among the 57 reports (I^2^ = 82.7%, *P* < 0.0001; Figure 2). The overall result demonstrated no statistically significant difference between cancer and normal control groups (SMD = −0.15, 95% CI: −0.35~0.04, *P* = 0.127; Figure 2, Table 2). Then, a subgroup analysis by cancer type was conducted. As shown in Figure 3 and Table 2, statistically significant differences between cancer and normal control groups were found for ESCA (SMD = −0.35, 95% CI: −0.5~-0.20, *P* < 0.0001; Figure 3A), PC (SMD = 0.45, 95% CI: 0.2~0.71, *P* = 0.001; Figure 3B) and HCC (SMD = −0.47, 95% CI: −0.60~-0.34, *P* < 0.0001; Figure 3C). The results suggested that NEAT1 was remarkably down-regulated in ESCA and HCC, while in PC, the expression level of NEAT1 was higher in cancer samples than that in normal specimens. However, the results for GC (SMD = −0.14, 95% CI: −0.75~0.46, *P* = 0.648, Figure 4A, Table 2), CRC (SMD = −0.12, 95% CI: −0.27~0.02, *P* = 0.086, Figure 4B, Table 2) and BTC (SMD = 0.02, 95% CI: −0.94~0.98, *P* = 0.971, Figure 4C, Table 2) demonstrated no statistically significant differences between cancer and normal control groups. We did not perform a subgroup analysis for GBC because there was only one gallbladder carcinoma-related dataset included in our research. The SMD of the one GBC-related record was −0.72 (95% CI: −2.4~0.96, *P* = 0.4).

**Table 2:** Pooled results of NEAT1 expression in digestive system cancers

				Heterogeneity		
Group	Number of datasets	SMD (95%CI)	*P* value	I^2^ (%)	*P* value	Model
Overall result	57	−0.15 (−0.35-0.04)	0.127	82.7	*P* < 0.0001	Random-effects model
ESCA	6	−0.35 (−0.50~-0.20)	*P* < 0.0001	46.0	0.099	Fixed-effects model
GC	8	−0.14 (−0.75~0.46)	0.648	85.4	*P* < 0.0001	Random-effects model
CRC	16	−0.12 (−0.27~0.02)	0.086	42.2	0.039	Fixed-effects model
PC	10	0.45 (0.20~0.71)	0.001	79.9	*P* < 0.0001	Random-effects model
HCC	13	−0.47 (−0.60~-0.34)	*P* < 0.0001	91.2	*P* < 0.0001	Random-effects model
BTC	3	0.02 (−0.94~0.98)	0.971	60.3	0.08	Random-effects model

SMD: standard mean deviation; CI: confidence interval

**Figure 5:**
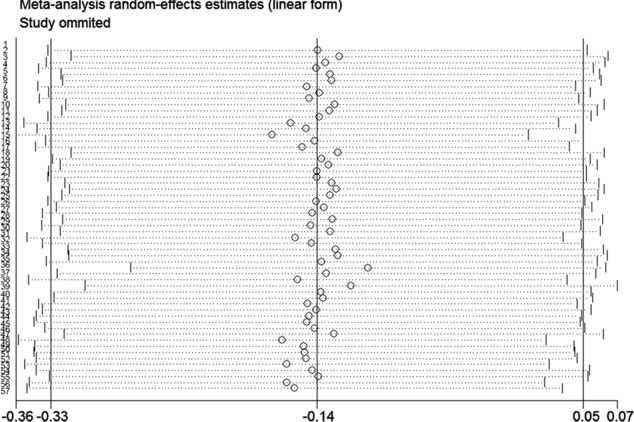
Result of the sensitivity analysis from a random-effect model The three vertical lines indicate the pooled SMD with 95% CI calculated from all included datasets. Each dotted horizontal line belongs to an independent study. In addition, the middle circle and two short vertical lines to the side represent the pooled SMD and its 95% CI corresponding to the successive exclusion of each dataset, respectively.

Furthermore, we carried out the sensitivity analysis by excluding individual datasets successively to evaluate the influence of each dataset on the pooled SMD. The result suggested that the pooled SMD was stable (Figure 5).

**Figure 6:**
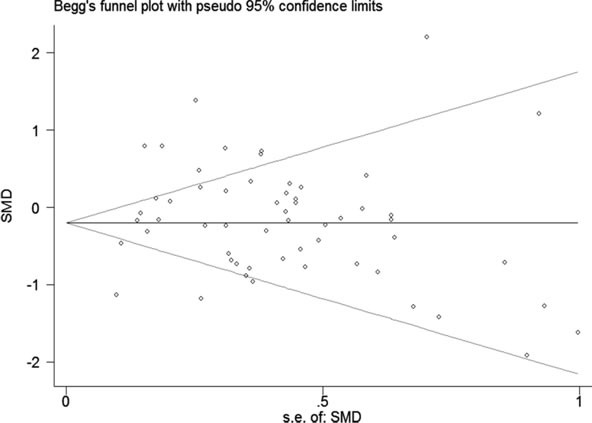
Funnel plot of the 57 included datasets The circles represent an individual dataset enrolled in the present investigation.

Additionally, a funnel plot was generated, and Begg's and Egger's tests were performed to assess the potential publication bias. The results showed that the funnel plot was nearly symmetric and the P values were greater than 0.05 (Begg's *P* = 0.12, Egger's *P* = 0.455; Figure 6), which indicated that there was no significant publication bias.

### Diagnostic role of NEAT1 in digestive system malignancies assessed by SROC

**Figure 7:**
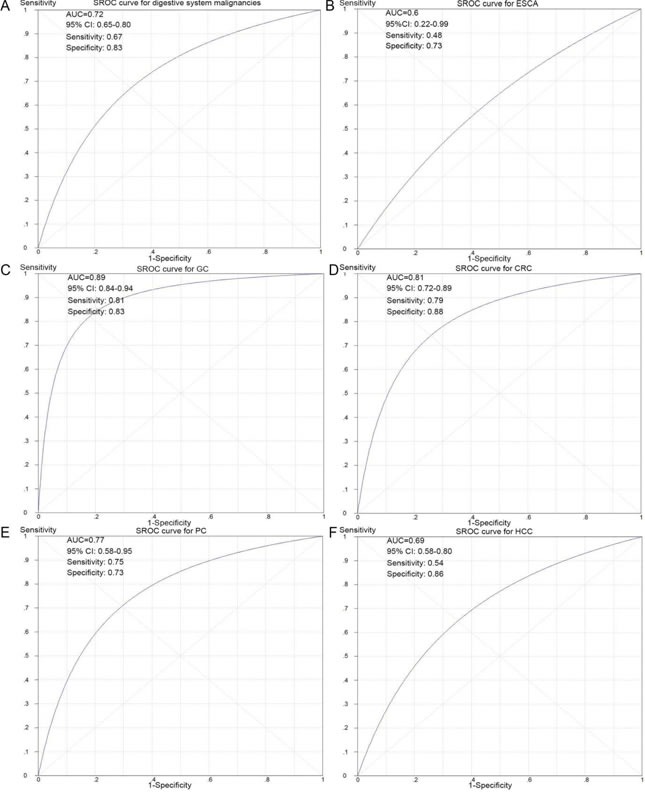
SROC curves for the differentiation of digestive system cancer patients from normal controls using NEAT1 expression **A**. The diagnostic ability of NEAT1 in all of the digestive system cancers. **B**. The diagnostic ability of NEAT1 in ESCA. **C**. The diagnostic ability of NEAT1 in GC. **D**. The diagnostic ability of NEAT1 in CRC. **E**. The diagnostic ability of NEAT1 in PC. **F**. The diagnostic ability of NEAT1 in HCC.

To further evaluate the diagnostic value of NEAT1 in digestive system carcinomas, we generated summary receiver operating characteristic (SROC) curves and then calculated the area under the curve (AUC) with the diagnostic sensitivity and specificity. As shown in Table 3 and Figure 7, the overall AUC of NEAT1 in digestive system cancers was 0.72 (95%CI: 0.65-0.80), and the diagnostic sensitivity and specificity were 0.67 and 0.83, respectively. Furthermore, the AUC values with 95% CIs of NEAT1 in ESCA, GC, CRC, PC and HCC were 0.6 (0.22-0.99), 0.89 (0.84-0.94), 0.81 (0.72-0.89), 0.77 (0.58-0.95) and 0.69 (0.58-0.80), respectively. Since there were only three BTC-related and one GBC-related lncRNA expression datasets available in this study, we did not generate SROC curves to assess the diagnostic role of NEAT1 in gallbladder carcinoma and biliary tract cancer.

**Table 3:** Diagnostic capability of NEAT1 in digestive system malignancies

		SROC			
**Cancer type**	**Number of datasets**	**AUC**	**95% CI**	**Sensitivity**	**Specificity**
Overall result	57	0.72	0.65-0.80	0.67	0.83
ESCA	6	0.6	0.22-0.99	0.48	0.73
GC	8	0.89	0.84-0.94	0.81	0.83
CRC	16	0.81	0.72-0.89	0.79	0.88
PC	10	0.77	0.58-0.95	0.75	0.73
HCC	13	0.69	0.58-0.80	0.54	0.86

AUC: area under the curve; SROC: summary receiver operating characteristic

### Prognostic value of NEAT1 in digestive system malignancies

To evaluate the association between NEAT1 expression and the prognosis of patients with digestive system cancers, we gathered the available published studies and the lncRNA-related microarray and RNA-seq datasets that included the information of prognostic value of NEAT1 in digestive system carcinomas. In total, five publications [22–26] and two microarray datasets with 977 cases were included, and the main information from the seven records (one record for GC, two records for ESCA, three records for CRC and one record for HCC) are summarized in Table 4.

**Table 4:** Characteristics of the seven prognosis-related published studies and microarray datasets

Data source	Test method/ Platform	Number of patients	Cancer type	Cut-off value	Outcome measurement	Analysis method	HR (95% CI)
PMID: 27095450	qRT-PCR	140	GC	NR	OS	Multivariate analysis	1.612 (1.026-2.532)
PMID: 26609486	qRT-PCR	96	ESCA	NR	OS	Multivariate analysis	1.919 (1.399-6.486)
PMID: 26314847	qRT-PCR	239	CRC	NR	OS DFS	Multivariate analysis	1.70 (1.18–2.45) 1.80 (1.27–2.55)
PMID: 26552600	qRT-PCR	191	CRC	NR	OS	Multivariate analysis	2.22 (1.23-4.00)
PMID: 26191242	qRT-PCR	95	HCC	5.9	RFS	Survival curve	1.93 (0.51-7.36)
GEO: GSE53625	GPL18109	179	ESCA	14.43	OS	Multivariate analysis	1.126 (0.86-1.476)
GEO: GSE31595	GPL570	37	CRC	11.79	OS	Multivariate analysis	0.848 (0.25-2.245)

NR: data not report; OS: overall survival; DFS: disease-free survival; RFS: relapse-free survival; HR: hazard ratio

**Figure 8:**
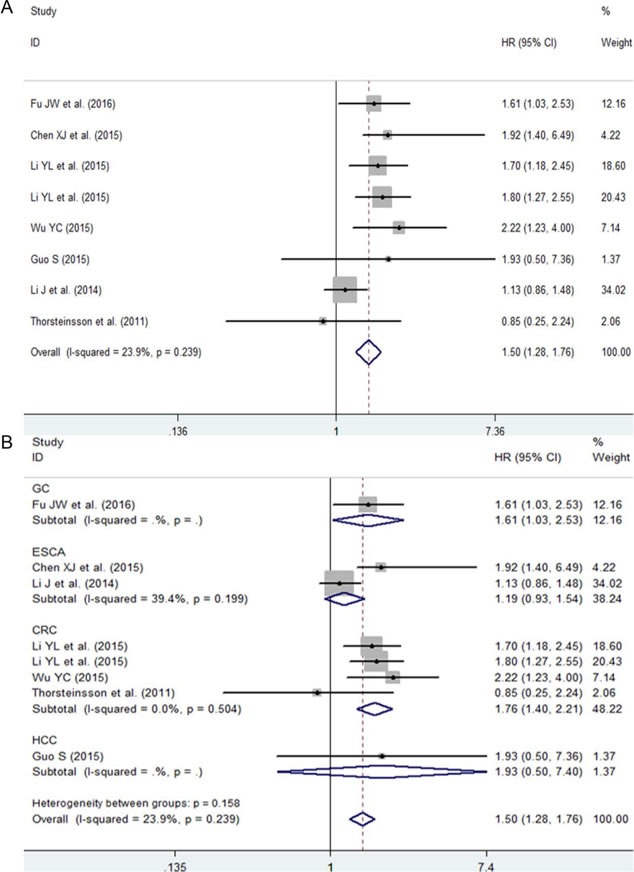
The prognostic role of NEAT1 in patients with digestive system malignancies **A**. Overall result with all of the available records. **B**. Subgroup analysis by cancer type. HR > 1 indicates a worse survival outcome for the group with an elevated NEAT1 expression. Each horizontal line represents an individual study. The middle point and the length of the two horizontal lines represent the HR and its 95% CI of each individual study, respectively. The diamond indicates the pooled HR and corresponding 95% CI. The middle vertical line is an invalid line.

The pooled hazard ratio (HR) from a fixed-effects model suggested that a high expression of NEAT1 was related to a poor survival outcome in patients with digestive system malignancies (HR: 1.50, 95% CI: 1.28-1.76, P < 0.0001; Figure 8A, Table 5). Additionally, a subgroup analysis by cancer type was also carried out. The results showed that an elevated expression of NEAT1 predicted an unfavorable prognosis in patients with CRC (HR: 1.73, 95% CI: 1.28-2.331, *P* < 0.0001; Figure 8B, Table 5). However, in patients with ESCA, no statistically significant difference between NEAT1 expression and prognosis was discovered (HR: 1.19, 95% CI: 0.53-1.54, *P* = 0.172; Figure 8B, Table 5). There was only one eligible study exploring the prognostic role of NEAT1 in GC and HCC, and the HRs were 1.61 (95% CI: 1.03-2.53, *P* = 0.038) and 1.93 (95% CI: 0.5-7.36, *P* = 0.338), respectively.

**Table 5:** The relationship between NEAT1 expression and prognosis in patients with digestive system carcinomas

					Heterogeneity	
Group	Number of records	Number of patients	HR (95%CI)	*P* value	I^2^ (%)	*P* value
Overall result	7	977	1.50 (1.28-1.76)	P<0.0001	23.9	0.239
ESCA	2	275	1.19 (0.93-1.54)	0.172	39.4	0.199
GC	1	140	1.61 (1.03-2.53)	0.038	none	none
CRC	3	467	1.73 (1.28-2.33)	P<0.0001	13.7	0.314
HCC	1	95	1.93 (0.50-7.40)	0.038	none	none

**Figure 9:**
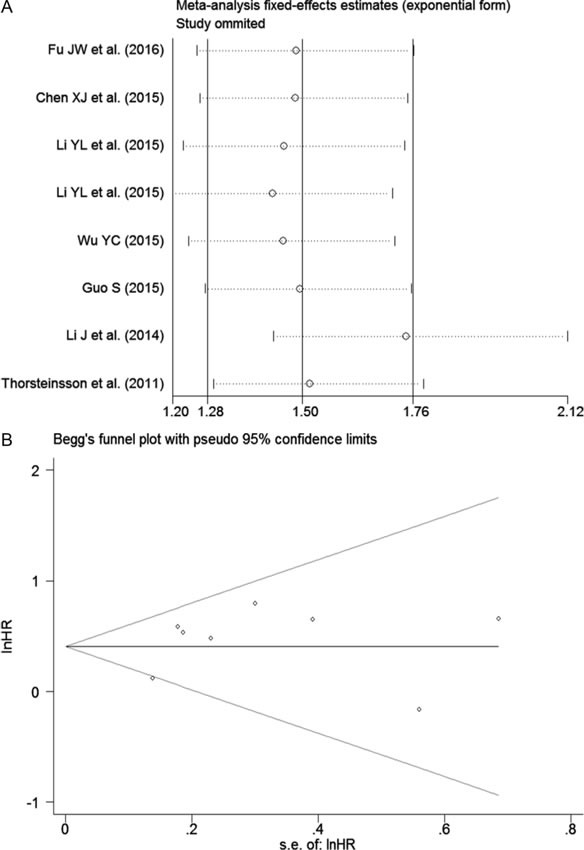
Results of the sensitivity analysis and publication bias **A**. Sensitivity analysis of HR (fixed-effects model), calculated by sequentially omitting each study. **B**. The funnel plot detects the potential publication bias among the seven included records.

Furthermore, a sensitivity analysis was performed, and that the result implied the pooled HR was stable (Figure 9A). In addition, Begg's and Egger's tests were carried out, and the *P* values were greater than 0.05 (Begg's *P* = 0.85, Egger's *P* = 0.81; Figure 9B), indicating that there was no significant publication bias.

## DISCUSSION

In recent years, countless studies have demonstrated that lncRNAs participate in various biological and chemical processes, such as cancer metastasis, chromosome remodeling, transcription and post-transcriptional processing [27]. Many studies have also proven that lncRNAs are related to carcinogenesis and the development of malignant tumors through varied pathways, including regulation of cell cycle [28, 29], apoptosis [30], autophagy [9], and chemotherapy resistance [31, 32] in cancer tissue or cell lines. LncRNAs have opened a completely novel field of cancer genomics.

NEAT1 is a long non-coding RNA that modulates gene expression and post-transcriptionally modifies primary transcripts [33]. Several studies have reported that NEAT1 is aberrantly expressed in different types of cancer, including in digestive system cancers. The dysregulation of NEAT1 may be associated with tumorigenesis and promote tumor progression [34–36].

Chen et al. [22] performed qRT-PCR on samples from 96 patients with ESCA to detect the expression level of NEAT1. The authors discovered that NEAT1 was highly expressed in tumor tissue, and an enhanced expression of NEAT1 stimulated the proliferation of ESCA cells and promoted their ability to form foci, migrate, and invade. However, our result derived from six microarray and RNA-seq datasets with 477 cancer patients and 344 normal controls showed that NEAT1 was down-regulated in ESCA tissues. Given the differences in total RNA extraction, NEAT1 expression level detection and sample sources, further rigorous studies with larger sample sizes are warranted to decipher the exact role of NEAT1 in ESCA.

Our research group previously performed qRT-PCR to detect NEAT1 expression in HCC tissue and matched paired non-cancerous tissue from 95 patients. The result revealed that NEAT1 had a higher expression level in HCC tissue than that in adjacent non-cancerous liver tissue; additionally, over-expressed NEAT1 promoted deterioration of HCC by influencing several clinicopathological characteristics, such as number of tumor nodes, infiltration, metastasis and clinical TNM stage [26]. However, Gibb et al. [37] obtained 272 serial analyses of gene expression (SAGE) libraries through the GEO and developed a lncRNA discovery pipeline to explore lncRNA expression in human cancer and normal tissue. They found that NEAT1 presented at a lower level of expression in liver cancer tissue than in normal tissue, which was consistent with our result based on the 57 microarray and RNA-seq datasets. Potential explanations for the discrepancies are the differences in sample sources and gene detection methods. Thus, further study is required to make a thorough inquiry of the role of NEAT1 in HCC.

In addition, according to our results, NEAT1 was more up-regulated in pancreatic cancer tissue than that in paired non-cancerous tissue, which demonstrated that NEAT1 might act as a cancer promoter and participate in the process of tumorigenesis of pancreatic cancer. However, there have been no published studies on the role of NEAT1 in PC. Given that our study was the first to identify this finding, further study is necessary to confirm our results.

In the present investigation, the expression levels of NEAT1 in GC and CRC cancer specimens were not significantly different from those of NEAT1 in non-cancerous samples. However, previous studies based on qRT-PCR showed that NEAT1 was over-expressed in GC and CRC. Fu et al. [23] measured the expression of NEAT1 in 140 GC samples and 4 gastric carcinoma cell lines by qRT-PCR and found that NEAT1 was up-regulated in both GC tissue and cell lines, played an important role in tumorigenesis and progression of GC and acted as a potential biomarker for diagnosis and prognosis. Ma et al. [38] also found that the expression of NEAT1 was elevated in gastric adenocarcinoma (GAC) patients, and a high expression of NEAT1 probably influenced GAC progression by promoting tumor growth. Li et al. [24] provided evidence that over-expressed NEAT1 may be an oncogene that could promote colorectal cancer differentiation, invasion and metastasis. We speculated that the diverse sample sources and detection methods were the main causes of the different conclusions between previous studies and our research.

Furthermore, we also evaluated the expression level of NEAT1 in BTC and GBC. Our results indicated that the expression levels of NEAT1 in cancer tissue were similar to those in adjacent non-cancer tissue. There have been no reports on the expression of NEAT1 in BTC and GBC as of yet, and the number of eligible datasets related to BTC and GBC in our study was only three and one, respectively. Thus further rigorous studies with more samples are warranted to explore the role of NEAT1 in gallbladder carcinoma and biliary tract cancer.

Our findings suggested that NEAT1 may have different expression patterns and play diverse roles in the initiation and development of different digestive system cancers. Our research results on the basis of microarray and RNA-seq datasets were not completely consistent with previous qRT-PCR based studies. On the one hand, the differences in total RNA extraction, NEAT1 expression level detection methods and sample sources are considered to be the potential explanations for these discrepancies. On the other hand, lncRNA NEAT1 has two variant: NEAT1_v1 and NEAT1_v2 [39]. The 3′-end processing mechanisms of NEAT1_v1 and NEAT1_v2 are distinct, which may lead to the different roles of these two transcripts in different types of cancer. The ratio of the two isoforms of NEAT1 may determine the trends for tumor development. Wu et al. [25] detected the expression of NEAT1_v1 and NEAT1_v2 in whole blood of colorectal cancer patients and found that both NEAT1_v1 and NEAT1_v2 were highly expressed in CRC. Then, the authors further evaluated the expression patterns of the two variants in colorectal cancer tissue and adjacent non-cancer tissue. They discovered that neither NEAT1_v1 nor NEAT1_v2 expression was significantly different between the tumor and normal tissue. The authors also demonstrated that the two transcripts predicted different clinical outcomes. A study conducted by Gao et al. [40] revealed that the expression level of NEAT1_v1 in leukemia samples was lower than those in normal specimens, while the expression level of NEAT1_v2 in leukemia was similar to those in normal controls. We assumed that the expression levels of the two isoforms were diverse in the different carcinomas. However, studies on the exact expression patterns and roles of NEAT1_v1 and NEAT1_v2 in digestive system malignancies are limited as of now. Therefore, more and larger studies are urgently needed to explore the exact significance of NEAT1_v1 and NEAT1_v2.

A previous study conducted by Wu et al. [25] demonstrated that NEAT1 could be a prospective diagnostic biomarker in CRC. However, the diagnostic performance of NEAT1 in other digestive system cancers has not yet been reported. Therefore, we performed this comprehensive investigation to explore the potential diagnostic value of NEAT1. According to our findings, NEAT1 presented a moderate diagnostic ability in digestive system malignancies. The present study was based on microarray and RNA-seq datasets; thus the sample size was large. However, the detection accuracy of gene chip technology may not be as precise as the qRT-PCR method. Thus, more reports based on qRT-PCR are indispensable in the clarification of the diagnostic capability of NEAT1.

Previous studies have proposed that NEAT1 could be a possible prognostic biomarker in cancer patients [22–25, 41]. A meta-analysis on the basis of published studies demonstrated that an increased expression of NEAT1 indicated a worse survival outcome in cancer patients [21]. The authors included 11 publications, including studies on eight types of neoplasm, to evaluate the prognostic role of NEAT1 in patients with carcinoma. However, in that meta-analysis, the relationships between NEAT1 expression and prognoses of digestive system malignancies had not been specifically proposed. Thus, we collected five available published studies and two microarray datasets involving 977 patients to comprehensively assess the correlations between NEAT1 expression and prognoses of patients with digestive system malignancies. In addition, our overall finding suggested an elevated expression of NEAT1 was related to a poor prognosis in patients with digestive system neoplasms. Further subgroup analyses indicated that NEAT1 could be a potential prognostic biomarker in CRC. Since only seven records with four cancer types (ESCA, GC, CRC and HCC) were identified in our research, more studies on the prognostic roles of NEAT in different types of digestive system cancers are needed to strengthen our conclusions.

**Figure 10:**
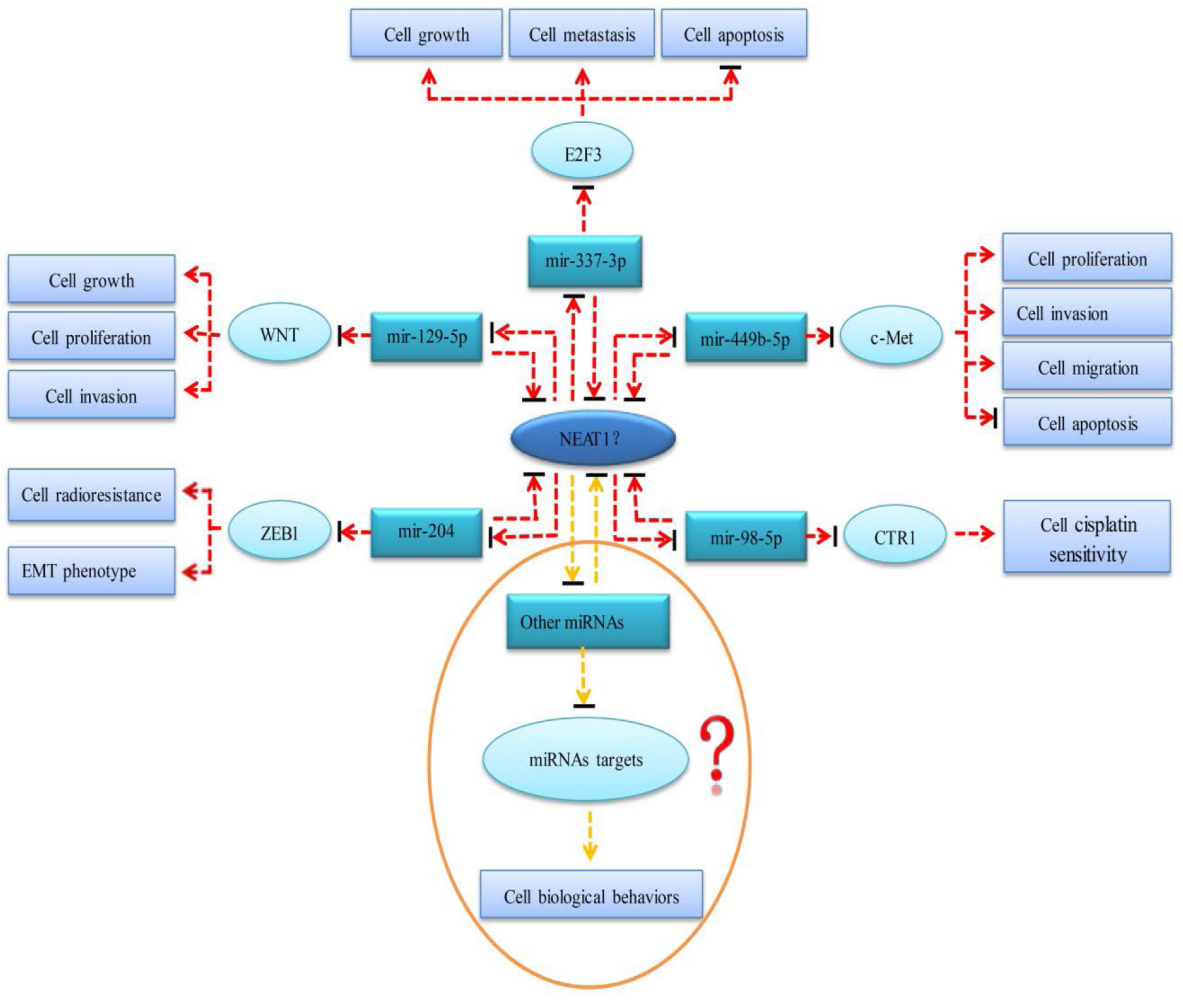
The prospective molecular mechanism of NEAT1 in the cancers of digestive system Red dotted lines represent mechanisms that have been verified in other tumors but not in digestive system malignancies. Yellow dotted lines represent potential mechanisms that have not been confirmed in any tumor. Arrows represent a promotion effect. Arrows combined with a short black line represent an inhibition effect.

According to our study, NEAT1 may play different roles in the initiation and progression of digestive system cancers. However, the molecular mechanism of NEAT1 in tumorigenesis and the development of digestive system malignancies is still limited and unclear. NEAT1 is an important component of paraspeckle, a subnuclear compartment that can regulate gene expression through a nuclear retention mechanism [42]. Therefore, NEAT1 probably affects the expression of certain tumor-related genes and further participates in the occurrence and evolvement of malignancies. Sun et al. [43] demonstrated that NEAT1 promoted the deterioration of non-small cell lung cancer (NSCLC) through negative modulation of mir-337-3p. NEAT1 may serve as a competitive endogenous RNA (ceRNA) and antagonize the inhibitory effect of mir-337-3p on oncogene E2F3. Lo et al. [36] proposed that NEAT1 could be inhibited by breast cancer susceptibility gene 1 (BRCA1) in breast cancer. BRCA1 is a tumor-suppressing gene that is located upstream of NEAT1. The authors also found that NEAT1 increased the malignant biological behaviors of BRCA1- knockdown breast neoplasm cells through suppressing mir-129-5p and subsequently enhancing the expression of oncogene WNT4. Zhen et al. [44] showed that NEAT1 played an ontogenetic role in gliomas by affecting the mir-449b-5p/c-Met axis. NEAT1 functioned as a miRNA sponge and thus relieved the inhibitory effect of mir-449b-5p on c-Met. Lu et al. [45] found that NEAT1 was over-expressed in nasopharyngeal carcinoma (NPC) tissue and cell lines. Up-regulation of NEAT1 promoted the expression of ZEB1 via inhibiting the activity of mir-204 and thus accelerated the deterioration of NPC. Additionally, the authors also silenced the NEAT1 gene and discovered that the down-regulation of NEAT1 reversed the epithelial to mesenchymal transition (EMT) phenotype and increased the radiosensitivity for NPC cells. NEAT1 is expected to be a potential therapeutic target of NPC. Another study conducted by Jiang et al. [46] suggested that NEAT1 upregulated CTR1 by sponging mir-98-5p and subsequently increased the cisplatin sensitivity of NSCLC cells. However, Gao et al. [40] found that NEAT1 was down-regulated in leukemia tissues and cell lines, serving as a tumor suppressor. After transfecting a NEAT1 plasmid into leukemia cell lines, the authors demonstrated that over-expression of NEAT1 could promote cell apoptosis and enhance the sensitivity of chemotherapy in leukemia cells. So far, there is no research on the mechanism of action of NEAT1 in digestive system tumors. We hypothesize that NEAT1 is involved in the occurrence and progression of digestive system cancers through ceRNA regulation networks. On the one hand, NEAT1 may act as a molecular sponge and repress the expression and biological functions of miRNAs, thereby reducing the inhibitory effects of miRNAs on their target genes. On the other hand, the competitive binding of NEAT1 and miRNAs may reverse the regulation of the expression and function of NEAT1. The prospective molecular mechanism of NEAT1 in the cancers of the digestive system is presented in Figure 10.

Certain limitations of the present study should be presented. First, all of the datasets were obtained from four online public databases of GEO, ArrayExpress, Oncomine and TCGA. On the one hand, the confounding factors induced by different RNA extraction methods and diverse RNA detection platforms may limit the validity of our meta-analysis result. On the other hand, the differences in the RNA detection methods and sample sources also resulted in a significant inter-study heterogeneity. Consequently, the random-effects model was selected to reduce the impact of the heterogeneity on our results. Second, in the present study, all of the samples were obtained from tumor and corresponding non-tumor tissue. However, a non-invasive diagnostic strategy is more valuable in the diagnosis of malignancies. Therefore, it would be of more clinical value to explore non-invasive diagnostic biomarkers derived from bodily fluid such as saliva and blood. Third, the number of eligible datasets in the present study was 57. However, there were only three and one datasets included for BTC and GBC, respectively. The small quantity of datasets and sample size may limit the stability of the pooled results. Hence, further well-designed researches including large sample size should be conducted to confirm NEAT1 expression level in digestive cancers. Fourth, there were only seven prognosis-related records with four types of cancer identified in the investigation. The pooled results may be unstable because the number of eligible studies was really small. Thus more reports on the prognostic role of NEAT1 in different types of digestive system cancers are necessary to validate our conclusion.

In summary, according to our results, NEAT1 might play different roles in the initiation and progression of digestive system cancers. In addition, more importantly, NEAT1 could be a prospective and valuable diagnostic and prognostic biomarker in patients with digestive system malignancies. However, the exact molecular mechanism of NEAT1 in tumorigenesis and the development of digestive system carcinomas remain unclear and needs to be explored further.

## MATERIALS AND METHODS

### Data acquisition

Digestive system cancer-related NEAT1 microarray and RNA-seq datasets were downloaded from the National Center of Biotechnology Information (NCBI) GEO (http://www.ncbi.nlm.nih.gov/geo/), ArrayExpress (http://www.ebi.ac.uk/arrayexpress/), Oncomine (https://www.oncomine.org/resource/main.html) and TCGA (http://cancergenome.nih.gov/). In addition, publications that referred to the prognostic value of NEAT1 in digestive system carcinomas were also retrieved from nine online databases: PubMed, Wiley Online Library, Web of Science, Cochrane Central Register of Controlled Trials, EMBASE, Chinese CNKI, Chong Qing VIP, Wan Fang and China Biology Medicine disc. The following search strategy was used: ((“NEAT1” OR “nuclear paraspeckle assembly transcript 1”) AND (“cancer” OR “tumor” OR “carcinoma” OR “neoplasm” OR “malignant” OR “malignancy”)). The retrieval date was up to December 31, 2016.

### Inclusion criteria

For digestive system cancer-related lncRNA microarray and RNA-seq datasets, eligible records were included if they met all of the criteria listed below: (1) study subjects within the cancer group were diagnosed with a digestive system cancer; (2) both cancerous samples and non-cancerous specimens were included in each dataset; (3) expression profiling data of NEAT1 were provided; and (4) the species included in the study were humans.

For published literature related to the prognostic value of NEAT1 in digestive system carcinomas, reports that fulfilled the following inclusion criteria were selected: (1) study objects must be human beings, and patients must be confirmed pathologically; (2) studies must evaluate the relationship between NEAT1 expression and prognosis in patients with digestive system cancers; and (3) Hazard ratios (HRs) and 95% confidence intervals (CIs) must be provided directly or could be estimated through sufficient survival data.

### Data extraction

Two researchers (Dan-dan Xiong and Zu-yun Li) independently collected information from all eligible datasets and published studies according to our inclusion criteria. Disagreements were determined through discussion with a third and fourth investigator (Zhen-bo Feng and Gang Chen). For lncRNA microarray and RNA-seq datasets correlated with the expression level of NEAT1, the following relevant data were extracted: first author and publication year, country, cancer type, sample source, data source, platform, expression values of NEAT1 and sample size in both cancer and normal control groups. If multiple probes were used, the maximum value of the probes was regarded as the expression value of NEAT1. For records referring to the prognostic role of NEAT1, the following main information was also collected: first author and publication year, region, data source, test method/platform, number of patients, cancer type, cut-off value, outcome measurement, analysis method and HR with its 95% CI. The most complete study was selected when the same patients were reported in different studies.

### Statistical analysis

All high-throughput expression data were log2-transformed. The mean and standard deviation were calculated using SPSS 20.0 (IBM, New York, USA) to estimate the expression level of NEAT1 in each of the datasets. Then, the overall SMD with 95% CI was evaluated using STATA, version 12.0 (StataCorp, College Station, TX, USA). An observed SMD>0 and its 95% CI not crossing zero indicated that NEAT1 had a higher expression level in cancerous specimens than that in non-cancerous samples.

To investigate the potential diagnostic performance of NEAT1 in digestive system malignancies, we generated SROC curves and calculated the AUC values with 95% CIs and the corresponding sensitivity and specificity using Meta-DISc software [47]. An AUC value of 0.5~0.7 represented a low diagnostic capability; an AUC of 0.7~0.9 indicated a moderate diagnostic ability; an AUC value of over 0.9 suggested a high diagnostic accuracy.

Additionally, we extracted HRs with corresponding 95% CIs directly if they were reported in an individual study; otherwise, we calculated them using a multivariate cox analysis based on the expression level of NEAT1 or extracted them using Engauge Digitizer Version 4.1 on the basis of the Kaplan-Meier survival curves. Then, we computed the pooled HR using STATA 12.0 to assess the prognostic significance of NEAT1 in patients with digestive carcinomas. A pooled HR over 1 and its 95% CI not crossing 1 indicated that an increase in the expression of NEAT1 predicted an unfavorable outcome.

Heterogeneity across studies was assessed using Cochran's Q [48] and I^2^ statistics [49]. A P value < 0.05 or I^2^>50% was considered to be heterogeneous, in which a random-effects model (DerSimonian-Laird method) was employed for pooling data [47]. Otherwise, a fixed-effects model (Mantel-Haenszel method) was utilized.

If significant heterogeneity was identified, subgroup analyses were carried out to further explore the heterogeneity source. Furthermore, a sensitivity analysis was conducted by omitting individual studies successively to evaluate the stability of the present meta-analysis [50]. Finally, we tested the publication bias by using a funnel plot with Begg's and Egger's bias indicator tests [51]. In addition, P < 0.05 indicated the presence of publication bias.
